# Supplementary figures and images for: Loss of miR-1469 expression mediates melanoma cell migration and invasion
Source: PLoS One. 2021 Sep 1;16(9):e0256629. doi: 10.1371/journal.pone.0256629 (PMC8409617; doi:10.1371/journal.pone.0256629)

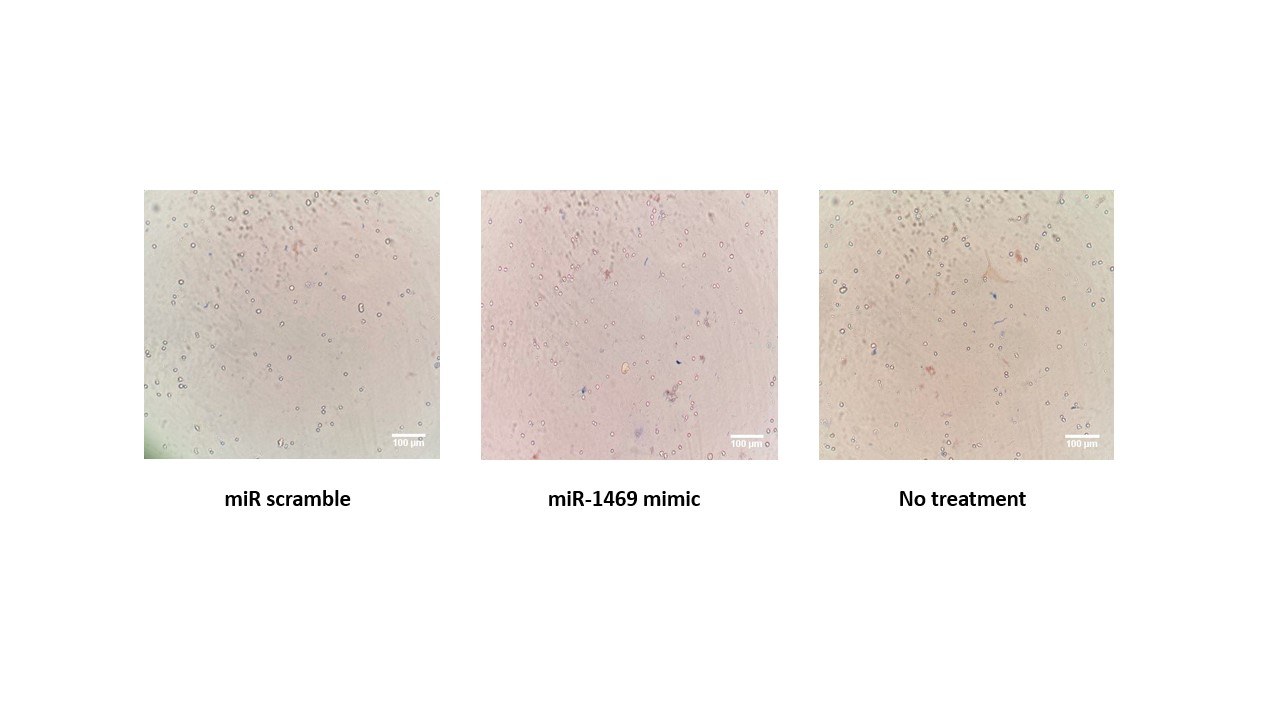

Supplement: S1 Fig — (TIF) [file pone.0256629.s001.tif]

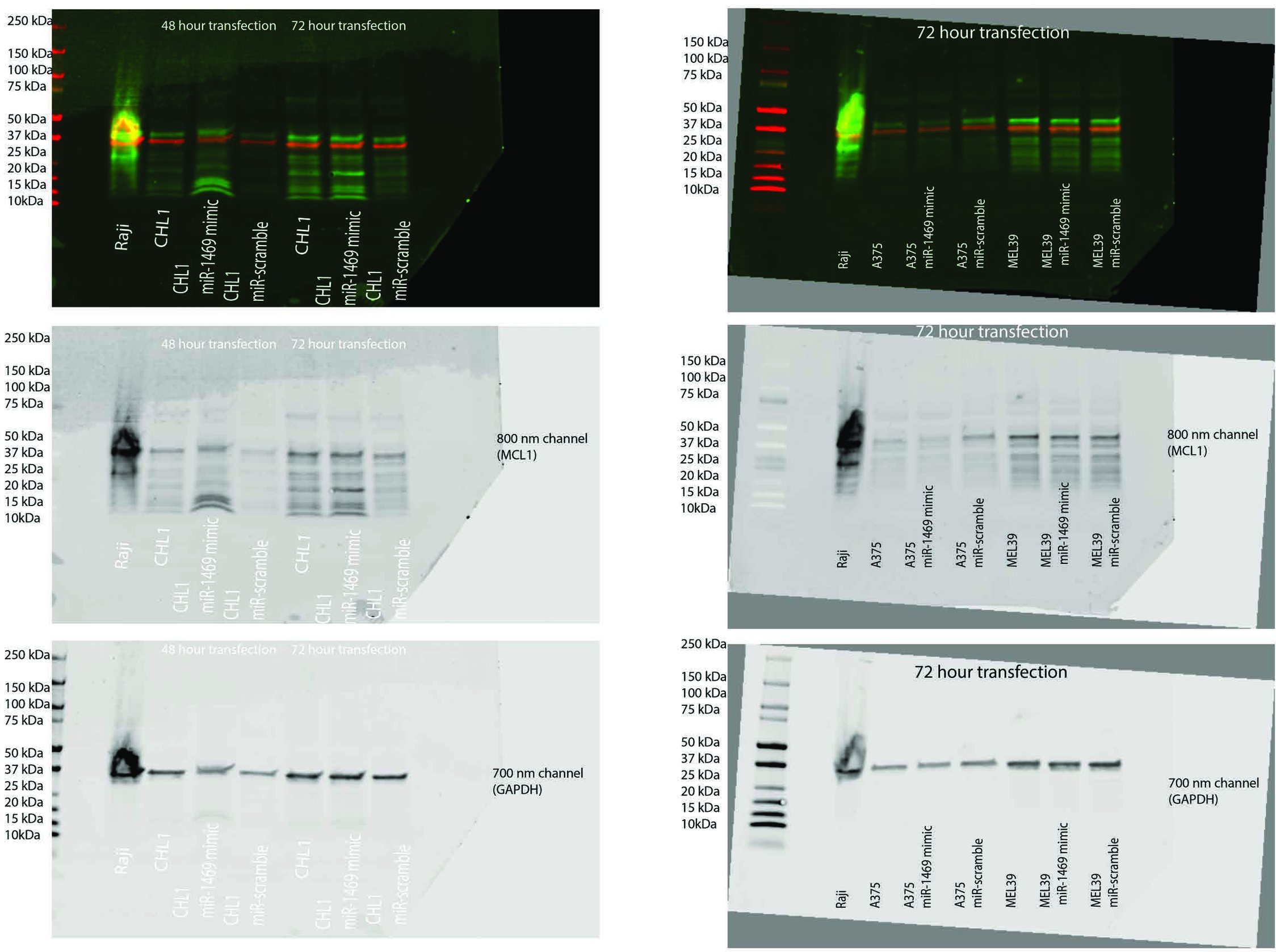

Supplement: S2 Fig — (TIF) [file pone.0256629.s002.tif]
